# Role of GPX4-Mediated Ferroptosis in the Sensitivity of Triple Negative Breast Cancer Cells to Gefitinib

**DOI:** 10.3389/fonc.2020.597434

**Published:** 2020-12-23

**Authors:** Xiang Song, Xinzhao Wang, Zhaoyun Liu, Zhiyong Yu

**Affiliations:** Department of Breast Cancer Center, Shandong Cancer Hospital and Institute, Shandong First Medical University and Shandong Academy of Medical Sciences, Jinan, China

**Keywords:** triple negative breast cancer, gefitinib, GPX4, ferroptosis, sensitivity

## Abstract

Gefitinib resistance in triple negative breast cancer (TNBC) is a growing important concern. Glutathione peroxidase 4 (GPX4) is a main regulator of ferroptosis, which is pivotal for TNBC cell growth. We investigated GPX4-mediated ferroptosis in gefitinib sensitivity in TNBC. Gefitinib resistant TNBC cells MDA-MB-231/Gef and HS578T/Gef were constructed and treated with lentivirus sh-GPX4 and ferroptosis inhibitor ferrostatin-1. GPX4 expression, cell viability and apoptosis were detected. Malondialdehyde (MDA), glutathione (GSH), reactive oxygen species (ROS) levels were evaluated. The levels of ferroptosis-related proteins were detected. Subcutaneous tumor model was established in nude mice, and gefitinib was intraperitoneally injected to evaluate tumor growth, apoptosis, and Ki-67 expression. GPX4 was increased in gefitinib-resistant cells. After silencing GPX4, the inhibition rate of cell viability was increased, the limitation of colony formation ability was reduced, apoptosis rate was increased, and the sensitivity of cells to gefitinib was improved. After silencing GPX4, MDA and ROS production were increased, while GSH was decreased. Silencing GPX4 promoted ferroptosis. Inhibition of GPX4 promoted gefitinib sensitivity by promoting cell ferroptosis. *In vivo* experiments also revealed that inhibition of GPX4 enhanced the anticancer effect of gefitinib through promoting ferroptosis. Overall, inhibition of GPX4 stimulated ferroptosis and enhanced TNBC cell sensitivity to gefitinib.

## Introduction

Breast cancer (BC) is the most common female tumor and leading cause of death among women (1). Triple-negative breast cancer (TNBC) accounts for about 20% of BC cases (2). TNBC is a distinct subtype of BC characterized by aggressive phenotype, high recurrence rates, high visceral metastasis rate, and worse outcomes (3–5). Molecular heterogeneity is prominent in the TNBC subtype, and is reflected by the obvious prognostic and patient’s sensitivity to chemotherapy treatment, which is the only available systemic therapy currently (1). Although TNBC patients benefit from standard chemotherapy, they still face a high recurrence rate and a high possibility of drug resistance, leaving TNBC a major challenge in the clinic (4, 6). Clinical trial data show that gefitinib is well tolerated in patients with a wide range of tumor types (7). Gefitinib, an epidermal growth factor receptor (EGFR) tyrosine kinase inhibitor, has shown both anti-proliferative and anti-tumoral activity in BC (8, 9). However, MDAMB-231 cells are resistant to clinically relevant doses of geftinib (10). Therefore, an understanding of the cellular targets of gefitinib will allow the discovery of biomarkers for predicting outcomes and provide information for overcoming gefitinib resistance in TNBC.

Targeting EGFR kinase activity by gefitinib insufficiently inhibits TNBC cell proliferation (11). Therefore, some synergistic interactions with other genes or drugs are paramount. Ferroptosis is a distinct type of cell death from the traditional apoptosis and necrosis, which is an iron-dependent cell death initiated and progressed by lipid peroxide accumulation and reactive oxygen species (ROS) production (12, 13). Ferroptosis is implicated in various diseases, including neurodegenerative diseases and cancers (14). Additionally, siramesine, a lysosome disrupting agent, and lapatinib, an EGFR inhibitor, elicit ferroptosis in a synergistic manner in BC cell lines by altering iron regulation (15). TNBC cells are reportedly sensitive to erastin-induced ferroptosis (16).

Furthermore, ferroptosis is specifically activated by missing glutathione peroxidase 4 (GPX4) activities (17). GPX4, an antioxidant defense enzyme is functional to repair oxidative damage to lipids and is a leading inhibitor of ferroptosis (18). Ferroptosis is featured with GSH depletion, disrupted GPX4 redox defense, inflammation, and detrimental lipid ROS formation (19, 20). However, whether GPX4-mediated ferroptosis is promising for gefitinib resistance in TNBC has not been reported. Therefore, we determined the role of GPX4-mediated ferroptosis in the mechanism of gefitinib resistance in TNBC cells, which could provide a new theoretical basis for the protective effects of GPX4 and contribute to developing effective drugs for TNBC in the future.

## Methods

### Treatment of Gef-Resistant Cells

Human TNBC cell lines MDA-MB-231 (ATCC^®^ CRM-HTB-26™) and HS578T (ATCC^®^ HTB-126™) (ATCC, VA, USA) were cultured in Dulbecco’s modified eagle’s medium (DMEM, 30-2002, ATCC) at 37℃ with 10% fetal bovine serum (FBS, 16000044, GIBCO, NY, USA). In order to construct Gef-resistant cells, MDA-MB-231 and HS578T cells were cultured in the medium with increasing concentration of gefitinib for 11 months [the highest concentration of gefitinib was 3 μM (21)]. Finally, the Gef-resistant cells MDA-MB-231/Gef and HS578T/Gef were stored in the medium containing 3 μM gefitinib (22). Gefitinib was purchased from MedChemExpress (HY-50895, Shanghai, China).

The Gef-resistant cells were assigned into sh-NC group (treated with lentivirus small hairpin RNA-negative control), sh-GPX4 group (treated with lentivirus sh-GPX4), sh-NC + DMSO group (treated with sh-NC + dimethyl sulphoxide), sh-GPX4 + DMSO (treated with lentivirus sh-GPX4 + DMSO), and sh-GPX4 + Ferrostatin-1 group (treated with lentivirus sh-GPX4 and 1 μM Ferrostatin-1) (23). Ferrostatin-1 is an inhibitor of ferroptosis and purchased from MedChemExpress (HY-100579). The titer of lentivirus was 1× 10^8^ Tu/ml.

### Reverse Transcription Quantitative Polymerase Chain Reaction (RT-qPCR)

Total RNA was extracted using RNeasy Mini Kit (Qiagen, Valencia, CA, USA) and reverse transcribed into cDNA using the reverse transcription kit (RR047A, Takara, Japan). RT-qPCR was performed using SYBR^®^ Premix Ex Taq™ II (Perfect Real Time) kit (DRR081, Takara, Japan) and real-time fluorescent qPCR instrument (ABI 7500, ABI, Foster City, CA, USA). The amplification procedures were pre-denaturation at 95°C for 30 s, and 40 cycles of 95°C 5 s, and 60°C 34 s. Each sample is provided with three duplicated wells. The primers were synthesized by Sangon Biotech (Shanghai) Co., Ltd. (Shanghai, China) (**Table 1**). Using GAPDH as an internal parameter, the relative expression of GPX4 was calculated by 2^-ΔΔCT^ method (24).

**Table 1:** Primer sequences for RT-qPCR.

	Forward Primer (5′-3′)	Reverse Primer (5′-3′)
GPX4	AGAGATCAAAGAGTTCGCCGC	TCTTCATCCACTTCCACAGCG
ACSL4	ATGAAACTTAAGCTAAATGTG	TTATTTGCCCCCATACATTCG
PTGS2	ATGCTCGCCCGCGCCCTGCTGC	CATTAGACTTCTACAGTTCAG
NOX1	ATGGGAAACTGGGTGGTTAAC	CCTATAACTCAAAAATTTTCTT
FTH1	GCCGCCATGACGACCGCGTCC	GCCCGAGGCTTAGCTTTCATTA
GAPDH	GGGAGCCAAAAGGGTCATCA	TGATGGCATGGACTGTGGTC

### Western Blot Analysis

Total protein was extracted using strong radio-immunoprecipitation assay lysis buffer (Boster Biological Technology Co., Ltd, Wuhan, Hubei, China), and the protein concentration was detected using a bicinchoninic acid protein assay kit (Boster). The protein was separated *via* 10% electrophoresis, transferred to polyvinylidene fluoride membranes, and blocked with 5% bovine serum albumin for 2 h to block the nonspecific binding. Afterward, the membranes underwent an overnight incubation with diluted primary antibodies GPX4 (ab125066, 1:2,000, Abcam, MA, USA), Ki-67 (ab16667, 1:1,000, Abcam), proliferating cell nuclear antigen (PCNA) (ab92552, 1:5,000, Abcam), Cleaved caspase-3 (ab2302, 1:1,000, Abcam), Cleaved caspase-9 (ab2324, 1:1,000, Abcam), acyl-CoA synthetase 4 (ACSL4) (ab155282, 1:10,000, Abcam), cyclooxygenase-2 (PTGS2) (ab15191, 1:1,000, Abcam), nicotinamide-adenine dinucleotide phosphate (NADPH) oxidase 1 (NOX1) (ab55831, 1:1,000, Abcam), ferritin heavy chain 1 (FTH1) (ab75972, 1:2,000, Abcam), and rabbit polyclonal β-actin (ab8227, 1:2,000, Abcam) at 4°C. Following washings, the membranes were incubated for 1 h with horseradish peroxidase (HRP)-labeled goat anti-rabbit secondary antibody immunoglobulin G (ab205718, 1:2,000, Abcam). The protein was developed using an enhanced chemiluminescence working solution (Millipore, Billerica, MA, USA). Image Pro Plus 6.0 (media cybernetics, USA) was used to quantify the gray level of each band, and β-actin was used as an internal reference. Each experiment was repeated three times (25).

### Cell Counting Kit-8 (CCK-8) Assay

After 48 h of transfection, the cells were seeded in 96-well plates at 0.4×10^4^ cells/well. Then the cells were treated with different doses of gefitinib (0, 2, 4, 6, 8, and 10 μM) for 48 h. CCK-8 assay kit (HY-K0301, MedChemExpress) was used to detect cell viability at 450 nm. The average inhibition rate of cell activity at each concentration was calculated (22, 23).

### Colony Formation Assay

After 48 h of transfection, 1,000 cells were seeded in 24-well plates. As previously described (26), the colonies were observed by crystal violet staining after 2 weeks of culture.

### Flow Cytometry

Annexin V FITC/PI double staining was utilized for apoptosis detection. After 48 h of transfection, cells were adjusted to 1×10^6^ cells/well. Cells were fixed overnight with 70% precooled ethanol solution at 4°C, and then 100 μl cell suspension (no less than 10^6^ cells/ml) was resuspended in 200 μl binding buffer. Then, the cells were stained with 10 μl Annexin V-FITC and 5 μl PI for 15 min in the dark. After the addition of 300 μl binding buffer, apoptosis at 488 nm was measured by flow cytometry, with 2×10^4^ cells each time (27).

### Determination of Malondialdehyde (MDA) and Glutathione (GSH)

MDA level and GSH level were measured using the Lipid Peroxidation Assay Kit (ab118970, Abcam) and the Glutathione Assay Kit (CS0260, Sigma, St. Louis, MO, USA), respectively.

### Assessment of ROS

After 48 hours of transfection, the cells were seeded in 6-well plates. After 8 h, the cells were stained with 20 μM 2’,7’-dichlorofluorescein diacetates (Beyotime Biotechnology, Shanghai, China) in the dark at 37°C for 30 min. The ROS level in the cells was observed by a fluorescence microscope (28).

### JC-1 Mitoscreen Assay

At 24 h after transfection, mitochondrial membrane potential (MMP) assay kit with JC-1 (C2006, Beyotime) was used to detect the MMP. The cells were imaged with a fluorescence microscope (28).

### Determination of Intracellular Iron Concentration

According to the instructions of the manufacturer of the iron ion kit (MAK025, Sigma), the cells were added into the buffer for iron determination. After mixing, the cells were centrifuged at 4°C at 13,000 g for 10 min to obtain the supernatant. At 25°C, 50 μl supernatant and 50 μl buffer were incubated in 96-well microplate for 30 min. Then the mixture was incubated with 200 μl reagent mixture in the dark at 25°C for 30 min, and then the absorbance value at 593 nm was measured to calculate the iron ion level.

### Xenograft Tumors in Nude Mice

Six-week-old female BALB nude (nu/nu) mice were purchased from Hunan SJA laboratory animal Co., Ltd. (Changsha, China; n = 40) and kept in a specific pathological free environment. In order to induce tumor *in vivo*, BALB/C nude mice were randomly allocated into four groups: sh-NC, sh-NC + Gef, sh-GPX4, and sh-GPX4 + Gef, with 10 mice in each group. Stable transfected cell lines were constructed by lentivirus infection. About 2×10^6^ cells were suspended in 200 μl phosphate-buffered saline (PBS). These cells were injected subcutaneously into the right hind leg of nude mice. Seven days after subcutaneous injection, gefitinib (10 mg/kg, once every other day) was intraperitoneally injected into sh-NC + Gef group and sh-GPX4 + Gef group for 2 weeks. On the 28^th^ day of subcutaneous injection, all tumors were collected, and the following tests were carried out.

### TUNEL Staining

The tumor tissue was fixed with 4% paraformaldehyde, dehydrated and cleared, and then paraffin embedded. The tumor tissue was sliced into 5 μm sections for staining. After dewaxing, the tissue sections were treated with conventional histological methods. To block peroxidase in tissues, the samples were placed in ethanol solution containing 3% hydrogen peroxide for 15 min. After washing, the samples were incubated with protease K for 20 min. After washing, the slides were incubated with the reaction solution of TUNEL staining kit. Then samples were incubated with 2,4-diaminobutyric acid (DAB) solution for 15 min, washed with PBS, and stained with hematoxylin. Finally, cells with brown nuclei were assessed as TUNEL-positive cells (29, 30).

### Immunohistochemistry

The parraffined sections were deparaffinized and hydrated, and the endogenous peroxidase activity was quenched by incubation in 0.3% H_2_O_2_ at 37°C for 30 min. After washing with PBS, the tissue sections were boiled in 10 mmol/L citrate buffer (pH 6.0) at 100°C for 30 min. Once cooled to room temperature, sections were sealed with 5% normal goat serum at 37°C for 1 h, followed by an overnight incubation with Ki-67 (ab16667, 1:200, Abcam) at 4°C. After washing three times in PBS, the tissue sections were incubated with the secondary antibody IgG (ab205718; 1:2,000) at 37°C for 1 h. After washing in PBS for three times, the tissue sections were then incubated with HRP-bound streptomyces avidin (1:1,000) at 37°C for 45 min. After that, the sections were stained with freshly prepared DAB. All tissue sections were counterstained with hematoxylin. Finally, the stained sections were analyzed under Olympus BX51 microscope (31).

### Statistical Analysis

SPSS 21.0 (IBM Corp., Armonk, NY, USA) was applied for data analysis. The measurement data are expressed in the form of mean ± standard deviation. Firstly, the normal distribution and variance homogeneity tests were conducted. If the data conformed to the normal distribution and homogeneity of variance, then the *t* test was used for comparison analysis between two groups. One-way or two-way analysis of variance (ANOVA) was used for comparison analysis among multiple groups, followed by Tukey’s multiple comparisons test. If the data did not conform to the normal distribution or homogeneity of variance, the rank sum test was carried out. The *p <*0.05 meant statistically significant.

## Results

### Gefitinib Induced GPX4 Expression in Gef-Resistant TNBC Cells

**Figure 1:**
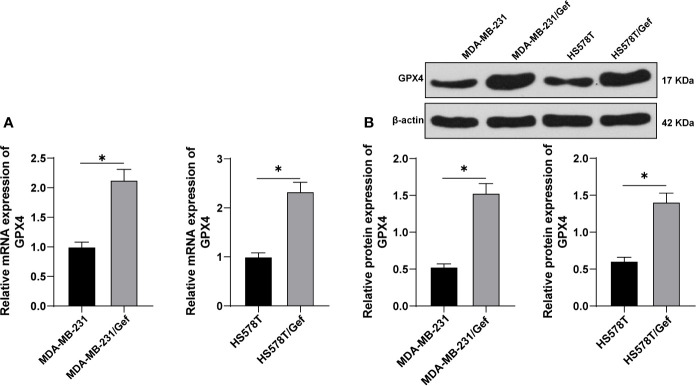
Gefitinib induced GPX4 expression in Gef-resistant TNBC cells. **(A, B)**, The levels of GPX4 were detected by RT-qPCR and Western blot. The cell experiment was repeated 3 times. The data were expressed as mean ± standard deviation and analyzed by the *t* test. *p < 0.05.

TNBC cell lines MDA-MB-231 and HS578T were selected to construct Gef-resistant strains. The levels of GPX4 were detected by RT-qPCR and Western blot. The results showed that the expression of GPX4 in the resistant strains was higher than that in the sensitive strains (**Figures 1A, B**) (*p* < 0.05). This indicated that gefitinib can induce the expression of GPX4 in TNBC cell lines MDA-MB-231 and HS578T.

### GPX4 Inhibition Increased Gefitinib Sensibility

**Figure 2:**
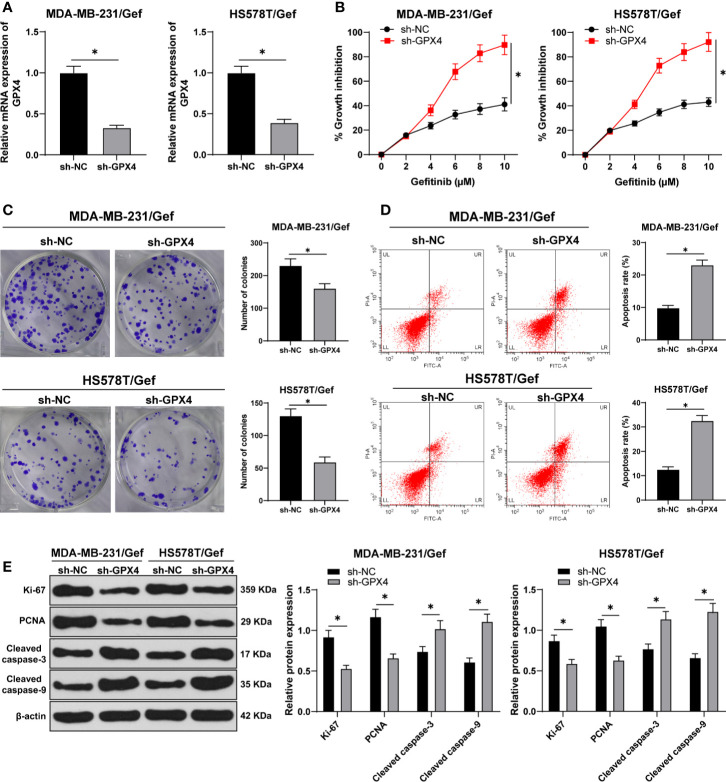
Inhibition of GPX4 can increase the sensitivity of TNBC cell lines to gefitinib. **(A)** The expression of GPX4 was detected by RT-qPCR; **(B)** CCK-8 was used to detect the inhibition rate of cell viability after different concentrations of gefitinib (0, 2, 4, 6, 8, 10 μm); **(C)** Colony formation assay was used to detect the ability of cell clone formation; **(D)** Flow cytometry was used to detect the apoptosis; **(E)** Western blot was used to detect the expression of Ki-67, PCNA, Cleaved caspase-3, and Cleaved caspase-9. The cell experiment was repeated 3 times. The data were expressed as mean ± standard deviation and analyzed by the *t* test (panels A/C/D) and two-way ANOVA (panels B/E), followed by Tukey’s multiple comparisons test. *p < 0.05.

To detect the relationship between GPX4 and gefitinib sensitivity, we silenced GPX4 in gefitinib resistant cell lines MDA-MB-231 and HS578T. RT-qPCR showed that GPX4 expression in sh-GPX4 group was lower than that in sh-NC group (**Figure 2A**). CCK-8 and colony formation assay were used to detect the proliferation of gefitinib resistant cell lines after silencing GPX4. With the increase of gefitinib concentration, the inhibition rate of cell activity increased significantly. Compared with sh-NC group, sh-GPX4 group significantly increased the cell activity inhibition rate (**Figure 2B**), and decreased cell colony formation (**Figure 2C**). Then we measured the apoptosis rate by flow cytometry (**Figure 2D**), and utilized Western blot to measure proliferation-related proteins Ki-67 and PCNA and apoptosis-related proteins Cleaved caspase-3 and Cleaved caspase-9 (**Figure 2E**) (all *p* < 0.05). Compared with sh-NC group, the apoptosis rate of sh-GPX4 group was clearly increased, the levels of Ki-67 and PCNA were significantly decreased, and the levels of Cleaved caspase-3 and Cleaved caspase-9 was increased in sh-GPX4 group. These results suggested that inhibition of GPX4 can increase the sensitivity of TNBC cell lines to gefitinib.

### GPX4 Negatively Regulated Ferroptosis in Gef-Resistant Cells

**Figure 3:**
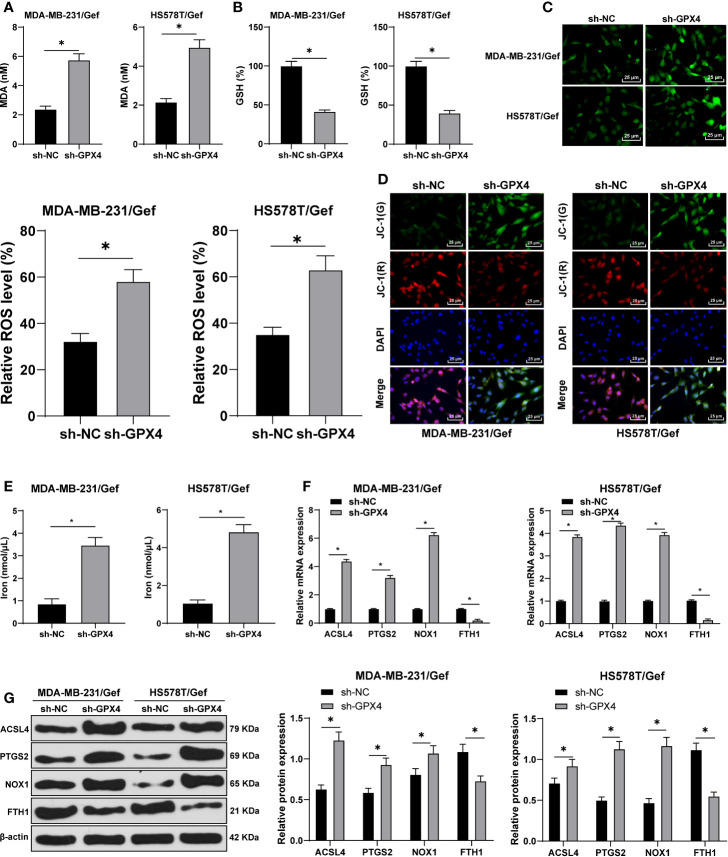
Inhibition of GPX4 can promote ferroptosis in gefitinib resistant cells. **(A)** MDA level in MDA-MB-231 and HS578T cells detected by a kit; **(B)** GSH level in MDA-MB-231 and HS578T cells detected by a kit; **(C)** ROS production was detected by fluorescence microscope (left) and fluorescence quantitative analysis (right), 400 ×, scale bar = 25 μM; **(D)** mitochondrial membrane potential test, 200 ×, scale bar = 50 μm; **(E)** the intracellular iron concentration was detected by iron ion kit; **(F, G)** RT-qPCR and Western blot detected the levels of ferroptosis-related proteins ACSl4, PTGS2, NOX1, and FTH1. The cell experiment was repeated 3 times. The data were expressed as mean ± standard deviation and analyzed by the *t* test (panels A/B/C) and two-way ANOVA **(E)** followed by Tukey’s multiple comparisons test. *p < 0.05.

Ferroptosis is very important for the survival of TNBC cells; ferroptosis is an iron dependent non apoptotic cell death, which is closely related to GPX4 (28). We first found that compared with sh-NC group, the MDA level was significantly increased after GPX4 silencing (**Figure 3A**), and GSH level was decreased (**Figure 3B**). Furthermore, we observed that ROS production increased significantly after GPX4 silencing (**Figure 3C**) and MMP reduced (**Figure 3D**). After that, we detected the intracellular iron concentration. Compared with sh-NC group, the intracellular iron concentration was significantly increased after GPx4 silencing (**Figure 3E**). We detected the levels of ferroptosis-related proteins ACSL4, PTGS2, NOX1, and FTH1 by Western blot. Compared with sh-NC group, the levels of ACSL4, PTGS2, and Nox1 were notably upregulated, while FTH1 levels were significantly downregulated in the sh-GPX4 group (**Figures 3F, G**) (all *p* < 0.05). The above results showed the same trend in MDA-MB-231/Gef and HS578T/Gef cells, indicating that inhibition of GPX4 can promote ferroptosis in gefitinib resistant cells.

### GPX4 Inhibition Increased Gefitinib Sensibility by Promoting Ferroptosis

**Figure 4:**
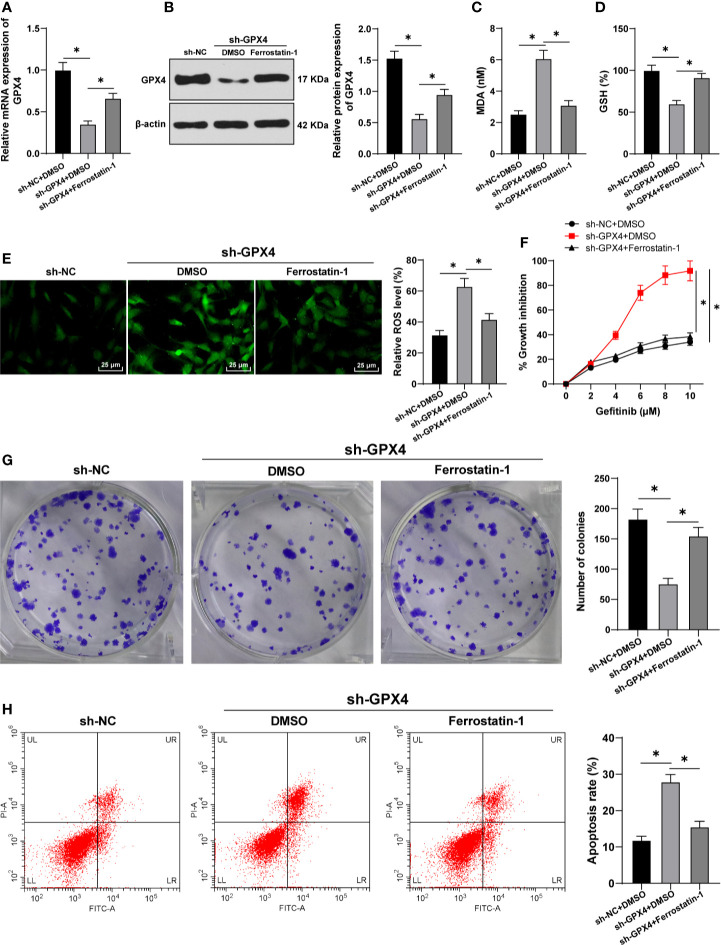
GPX4 inhibition increased gefitinib sensibility by promoting ferroptosis. While silencing GPX4 in TNBC cell line MDA-MB-231, ferrostatin-1 was used to inhibit ferroptosis. **(A, B)** The levels of GPX4 were detected by RT-qPCR and Western blot; **(C)** MDA level in MDA-MB-231 and HS578T cells detected by a kit; **(D)** GSH level in MDA-MB-231 and HS578T cells detected by a kit; **(E)** ROS production was detected by fluorescence microscope (left) and fluorescence quantitative analysis (right), 400 ×, scale bar = 25 μM; **(F)** CCK-8 was used to detect the inhibition rate of cell viability after different concentrations of gefitinib (0, 2, 4, 6, 8, 10 μm); **(G)** Colony formation assay was used to detect the ability of cell clone formation; **(H)** Flow cytometry was used to detect the apoptosis. The cell experiment was repeated 3 times. The data were expressed as mean ± standard deviation and analyzed by one-way ANOVA **(A–E, G–H)** and two-way ANOVA **(F)** followed by Tukey’s multiple comparisons test. *p < 0.05.

To further confirm that the regulation of gefitinib sensitivity by GPX4 is achieved by affecting ferroptosis, we silenced GPX4 in TNBC cell line MDA-MB-231 and used ferrostatin-1 to inhibit ferroptosis. GPX4 levels were detected by RT-qPCR and Western blot. The results showed that GPX4 levels were effectively reduced after silencing GPX4, but partially restored by ferrostatin-1 (**Figures 4A, B**). After silencing GPX4, MDA level increased significantly (**Figure 4C**), GSH level decreased (**Figure 4D**), and ROS production increased (**Figure 4E**) (all *p* < 0.05). However, inhibition of ferroptosis by ferrostatin-1 partially reversed the effect of sh-GPX4. These results further indicated that GPX4 can negatively regulate ferroptosis.

Then, we detected the cell activity by CCK-8 and colony formation assays. The inhibition rate of cell activity was clearly increased after silencing GPX4, while the effect of sh-GPX4 was partially reversed by ferrostatin-1 (**Figures 4F, G**). In addition, flow cytometry showed that the apoptosis rate was increased after silencing GPX4, and ferrostatin-1 partially reversed the promoting effect of sh-GPX4 on apoptosis (**Figure 4H**) (all *p* < 0.05). All these results suggested that inhibition of GPX4 promoted gefitinib sensitivity by promoting cell ferroptosis.

### GPX4 Inhibition Increased the Anti-Tumor Effect of Gefitinib by Promoting Ferroptosis

**Figure 5:**
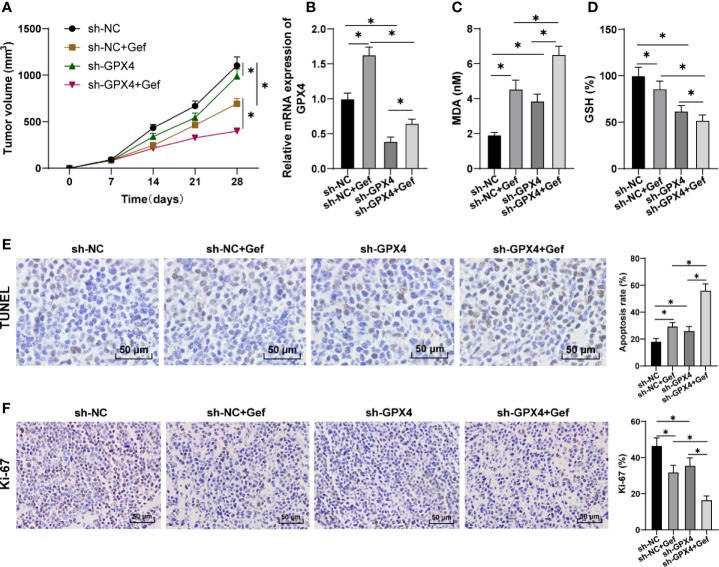
GPX4 inhibition increased the anti-tumor effect of gefitinib by promoting ferroptosis. **(A)** Tumor growth curve; **(B)** GPX4 expression was detected by RT-qPCR; **(C)** MDA level was detected by a kit; **(D)** GSH level was detected by a kit; **(E)** TUNEL staining was used to detect apoptosis, 400 ×, scale bar = 25 μm; **(F)** Immunohistochemistry was used to detect the expression of Ki-67, 400 ×, scale bar = 25 μm; n = 10. The data were expressed as mean ± standard deviation and analyzed by one-way ANOVA **(B–F)** and two-way ANOVA **(A)** followed by Tukey’s multiple comparisons test. *p < 0.05.

After constructing the subcutaneous tumor model in nude mice, sh-GPX4 treatment significantly reduced the growth rate of tumor in nude mice, and further increased the inhibitory effect of gefitinib on tumor (**Figure 5A**). Gefitinib promoted GPX4 expression, while sh-GPX4 treatment effectively inhibited GPX4 expression (**Figure 5B**). After treatment with gefitinib alone or silencing GPX4 alone, MDA was significantly increased, while GSH was decreased. Silencing GPX4 could further enhance the regulation of gefitinib on MDA and GSH (**Figures 5C, D**). Finally, TUNEL staining and immunohistochemistry were used to detect the apoptosis and Ki-67 expression. The results showed that gefitinib alone or silencing GPX4 alone notably increased the apoptosis rate and decreased Ki-67 expression. After gefitinib treatment and silencing GPX4, the apoptosis rate was further increased, and Ki-67 expression was further decreased (**Figures 5E, F**). All in all, inhibition of GPX4 enhanced the anticancer effect of gefitinib *in vivo* by promoting cell ferroptosis.

## Discussion

Inhibition of ferroptosis is potential to facilitate sorafenib (an EGFR inhibitor) resistance to cancer cells (23, 32). Previous study has identified a potential therapeutic benefit of blocking EGFR with gefitinib in basal-like subtypes of TNBC *in vitro* (21). Besides, GPX4 is a promising target for killing therapy-resistant cancer cells *via* ferroptosis (33). However, the role of GPX4-mediated ferroptosis in gefitinib sensitivity in TNBC cells is largely unknown. Herein, this study focused on the possible mechanism of GPX4 in gefitinib sensitivity in TNBC cells by monitoring ferroptosis.

GPX4 is positively related to chemoresistance of anticancer drug lapatinib and IC50 of lapatinib (an inhibitor of EGFR) (34). In this study, we found that GPX4 expression in the resistant TNBC cell strains was higher than that in the sensitive strains. This indicated that gefitinib can induce the expression of GPX4 in TNBC cell lines MDA-MB-231 and HS578T. As reported, inhibition of tumor propellant GPX4 enhances anticancer effect of chemotherapeutic drugs (34). Then we silenced GPX4 expression in MDA-MB-231/Gef and HS578T/Gef cells to test its effect on gefitinib sensitivity. With the increase of gefitinib concentrations, the inhibition rate of cell activity was increased, and cell colony formation was decreased significantly. The apoptosis rate of sh-GPX4-treated cells was clearly increased, the levels of Ki-67 and PCNA were decreased, and the levels of Cleaved caspase-3 and Cleaved caspase-9 were increased. Similarly, in resistant cells overexpressing GPX4, GPX4 inhibitor overcomes resistance to erlotinib, and knockdown of GPX4 inhibits the migration of resistant cells (35). Decreased protein level of GPX4 is accompanied with increased levels of cleaved caspase 3 and iron concentrations (36). These results suggested that inhibition of GPX4 can increase the sensitivity of TNBC cell lines to gefitinib.

Increasing evidences have indicated that promoting ferroptosis is a promising approach to attenuate drug resistance in cancer chemotherapy (37, 38). Given the therapeutic promise for inducing ferroptosis in drug-resistant cancers, a potent GPX4 inhibitor is paramount (39, 40). Inhibition of GPX4 leads to the induction of ferroptosis (41), especially in drug-resistant tumors (42, 43). After GPX4 silencing, our data revealed that MDA level was notably increased, and GSH level was decreased, ROS production was increased significantly and MMP was reduced. Ferroptosis is very important for the survival of TNBC cells and is closely related to GPX4 (28). Lipid ROS increases after suppression of GPX4 (32). Elimination of ROS sensitizes the BC cells to gefitinib (44). GSH is the most decreased cellular metabolite during ferroptosis, and GSH depletion causes loss of cellular antioxidant and inhibition of GPXs (13, 45). Ferroptosis induces GSH depletion, disrupted GPX4 function and ROS production (14, 19, 46). Moreover, the levels of ACSL4, PTGS2 and NOX1 were notably upregulated, while FTH1 was downregulated in the sh-GPX4 group. ACSL4 is preferentially expressed in TNBC cell lines and cells with GPX4-ACSL4 double knockout show marked resistance to ferroptosis (47). ACSL4 inhibition is a viable therapeutic approach to preventing ferroptosis-related diseases (48). The above results showed the same trends in MDA-MB-231/Gef and HS578T/Gef cells, indicating that inhibition of GPX4 can promote ferroptosis in gefitinib resistant cells.

To further confirm that the regulation of gefitinib sensitivity by GPX4 is achieved by affecting ferroptosis, we silenced GPX4 in TNBC cell line MDA-MB-231 and used ferrostatin-1 to inhibit ferroptosis. GPX4 expression was effectively reduced after silencing GPX4, but partially restored by ferrostatin-1. Consistently, ferrostatin-1 distinctly raises the GPX4 and FTH1 expression and blocks the PTGS2 and ACSL4 level (49). After silencing GPX4, MDA level was increased, GSH level was decreased, and ROS production was increased, and apoptosis rate was elevated. However, inhibition of ferroptosis by ferrostatin-1 partially reversed the effect of sh-GPX4. The enhancement of ROS generation in H1650 and H1975 Gef-resistant cells leads to impairment of tumor growth and induction of cell apoptosis (50). In lung cancer cell lines, cell proliferation is decreased with GPX4 knockdown and this inhibition could be reversed by ferrostatin-1, the specific inhibitor of ferroptosis (14, 51). These results further indicated that GPX4 can negatively regulate ferroptosis, and ferrostatin-1 partially reversed the effect of sh-GPX4. Furthermore, sh-GPX4 treatment reduced the growth rate of tumor in nude mice, and further strengthened the inhibitory effect of gefitinib on tumors. All in all, inhibition of GPX4 enhanced the anticancer effect of gefitinib *in vivo* by promoting cell ferroptosis.

In summary, this study simply revealed that GPX4 knockdown can enhance the sensitivity of TNBC cells to gefitinib by promoting cell ferroptosis, but the regulatory mechanism of GPX4 expression in gefitinib resistant cells has not been studied in depth. Due to the current funding and cycle limitations, we cannot study more inhibitors for GPX4 ablation in this paper. But we will apply more inhibitors for sh-GPX4 lines to ferroptosis research in the future, in order to provide more information on the mechanism of inhibiting lipid peroxidation. In the future, we will study the regulation mechanism of GPX4 expression in gefitinib resistant TNBC cells from the perspective of transcriptional regulation.

## Data Availability Statement

The original contributions presented in the study are included in the article/supplementary material. Further inquiries can be directed to the corresponding author.

## Ethics Statement

This study was reviewed and approved by the ethics committee of Shandong Cancer Hospital and Institute.

## Author Contributions

Conceptualization: XS and XW. Validation, research, resources, data reviewing, and writing: ZL. Review and editing: ZY. All authors contributed to the article and approved the submitted version.

## Conflict of Interest

The authors declare that the research was conducted in the absence of any commercial or financial relationships that could be construed as a potential conflict of interest.
